# Efficacy and Safety of Oral Betamethasone Mini-Pulses in Moderate to Severe Alopecia Areata

**DOI:** 10.7759/cureus.84000

**Published:** 2025-05-13

**Authors:** Rasha Moumna, Zineb Loubaris, Anas Ahmed Mountassir, Majdouline Obtel, Benzekri Laila, Mariame Meziane

**Affiliations:** 1 Dermatology, Ibn Sina University Hospital, Mohammed V University, Rabat, MAR; 2 Epidemiology and Public Health, Faculty of Medicine and Pharmacy of Rabat, Mohammed V University, Rabat, MAR

**Keywords:** alopecia areata (aa), alopecia areata (aa) treatment, betamethasone, corticosteroid treatment, pulse therapy, trichoscopy

## Abstract

Introduction

Alopecia areata (AA) is an acquired autoimmune disorder affecting hair follicles, responsible for non-scarring alopecia with an unpredictable clinical course. Systemic corticosteroids are commonly prescribed in acute or rapidly progressing forms, but their long-term use is limited by potential adverse effects. Intermittent oral corticosteroid regimens, referred to as mini-pulses, have emerged as a promising alternative.

Objectives

This study aims to assess the efficacy, safety, and sustainability of the response to oral betamethasone mini-pulses in patients with moderate to severe or refractory AA and to explore clinical and trichoscopic predictors of treatment response.

Materials and methods

We conducted a retrospective cohort study including 40 patients treated for AA at Ibn Sina University Hospital, Morocco, from 2022 to 2025. All patients received oral betamethasone mini-pulses at fixed doses (2 mg for children, 4 mg for adults), administered two consecutive days per week for at least three months, with treatment duration adjusted based on clinical response. Clinical, dermoscopic, and quality of life parameters were assessed using standardized tools, including the Severity of Alopecia Tool (SALT), the Alopecia Areata Scale (AAS), Eyebrow and Eyelash Assessment Scales (EBA/ELA), and the Dermatology Life Quality Index (DLQI). Predictors of treatment response and relapse were analyzed statistically.

Results

A ≥50% hair regrowth (SALT-50) was achieved in 62.5% of patients, including complete regrowth in 25%. Dermoscopic activity markers and DLQI scores also improved significantly after treatment. Adverse events occurred in 20% of cases; all were mild and transitory. Relapses were observed in 15% of patients, mostly partial. Better responses were significantly associated with patchy AA and lower baseline SALT scores. In contrast, poor response correlated with atopic diathesis, including asthma and allergic rhinitis, as well as rapidly progressive forms and higher initial SALT scores. Relapse was associated with autoimmune thyroiditis and vitamin D deficiency.

Conclusion

Oral betamethasone mini-pulse therapy represents an effective and well-tolerated treatment option for moderate to severe or refractory AA. It is particularly relevant in resource-limited settings where access to advanced therapies such as JAK inhibitors remains restricted. Larger, long-term studies are needed to validate these findings, refine treatment duration, and identify ideal responders.

## Introduction

Alopecia areata (AA) is a common autoimmune disease targeting the hair follicle, with multifactorial pathogenesis involving genetic, immunologic, and environmental contributors [1]. It usually presents as well-demarcated patches of non-scarring alopecia on the scalp. However, it can progress to more severe forms such as alopecia totalis (AT) or alopecia universalis (AU) and may also affect the eyebrows, eyelashes, body hair, and nails, contributing to its physical and psychosocial burden [1].

Systemic corticosteroids are a standard therapeutic option in acute or extensive forms of AA. However, their long-term use is limited by the relatively high risk of relapses and potential adverse effects [2]. To reduce these risks, intermittent oral corticosteroid regimens - commonly referred to as “mini-pulses” - have been proposed. These protocols typically involve administering corticosteroids two days per week to maintain efficacy while lowering cumulative toxicity [2]. However, oral corticosteroid mini-pulses for AA remain insufficiently studied and underdocumented in the literature [3]. Yet they represent a promising and affordable therapeutic approach, especially in resource-limited settings where access to advanced therapies such as Janus kinase (JAK) inhibitors is restricted.

This study aims to assess the efficacy and safety of oral betamethasone mini-pulse therapy in moderate to severe or refractory AA. Secondary objectives include evaluating the durability of regrowth after treatment cessation, identifying clinical and trichoscopic predictors of response, and comparing findings with existing literature.

## Materials and methods

Study design and setting

This was a retrospective cohort study with both descriptive and analytical approaches, conducted at the Dermatology Department of Ibn Sina University Hospital in Rabat, Morocco, over a three-year period (February 2022 to February 2025).

Inclusion and exclusion criteria

Patients were included if they were at least five years old, had a diagnosis of AA confirmed by clinical and dermoscopic findings, and had received oral betamethasone mini-pulses for at least three months.

Patients were excluded if they had ongoing or recent (<1 month) systemic treatment for AA other than betamethasone mini-pulses, incomplete clinical documentation or photographic records, or were lost to follow-up.

Treatment protocol

In this cohort, all patients had received oral betamethasone mini-pulses at fixed doses of 2 mg/day for children and 4 mg/day for adults, administered two consecutive days per week. Treatment tapering was performed at variable times, depending on when cosmetically acceptable regrowth was achieved. Daily zinc supplementation and topical 2% minoxidil had also been routinely prescribed, and iron and vitamin D supplementation were added when deficiencies were documented.

Outcome measures

Treatment efficacy was assessed using the Severity of Alopecia Tool (SALT) [4], the Alopecia Areata Scale (AAS) [5], the Eyebrow (EBA) and Eyelash (ELA) Assessment Scales [6], the Dermatology Life Quality Index (DLQI) [7], and a visual analog scale for patient satisfaction (ranging from 0: not satisfied at all to 10: highly satisfied). Trichoscopy was also used in all patients to monitor disease activity and assess response to treatment.

Data collection

Data were extracted from patient medical records and standardized clinical photographs. In cases of missing clinical information, data were collected through follow-up with the patient or their family.

Statistical analysis

Descriptive statistics were used to summarize patient characteristics. Quantitative variables were expressed as means ± standard deviation or medians with interquartile ranges, depending on the distribution. Categorical variables were expressed as counts and percentages.

Analytical comparisons of pre- and post-treatment outcomes were performed using the Wilcoxon signed-rank test. Baseline factors associated with treatment response and relapse were analyzed using the Mann-Whitney U test for continuous variables and Fisher’s exact test for categorical variables. A p-value less than 0.05 was considered statistically significant.

Ethical considerations

This study was conducted in accordance with the ethical principles of the Declaration of Helsinki [8]. As it was a retrospective study of anonymized patient records, formal ethical approval was not required according to our institutional policy. Informed consent was obtained from all patients or their legal guardians for the use and publication of their clinical data and photographs.

## Results

Patient demographics and comorbidities

A total of 40 patients (n = 40) were included, with a female predominance (65%). The mean age was 16.5 years (range: 6-50), with children and adolescents representing 70% of the cohort and adults representing 30%. Comorbid conditions included atopic diseases (25%), autoimmune thyroiditis (17.5%), and psychiatric manifestations such as anxiety or depression (10%). A family history of AA was reported in 12.5% of cases.

Disease baseline characteristics

An identifiable trigger was reported by 50% of patients. Psychological stress was the most frequent (35%), followed by COVID-19 vaccination, infection, or post-partum status (each 5%). In 30% of cases, consultation was delayed for more than one year after disease onset.

At baseline, the mean SALT score was 54.1%. According to the AAS, 50% of patients had severe forms (SALT ≥ 50), including 42.5% with AT/AU; 40% had moderate AA (SALT 21-49), and 10% had mild but refractory forms (SALT ≤ 20). Rapidly progressive AA (RP-AA), defined as diffuse hair loss with ≥50% scalp involvement within less than three months [9], was observed in 22.5% of cases. Eyebrow involvement (EBA 0-2) was observed in 37.5% of patients, eyelash loss (ELA 0-2) in 27.5%, and body hair involvement (B1-B2) in 20%. The mean baseline DLQI score was 5.2, with 40% reporting a moderate to severe impact on quality of life.

Treatment efficacy

**Figure 1:**
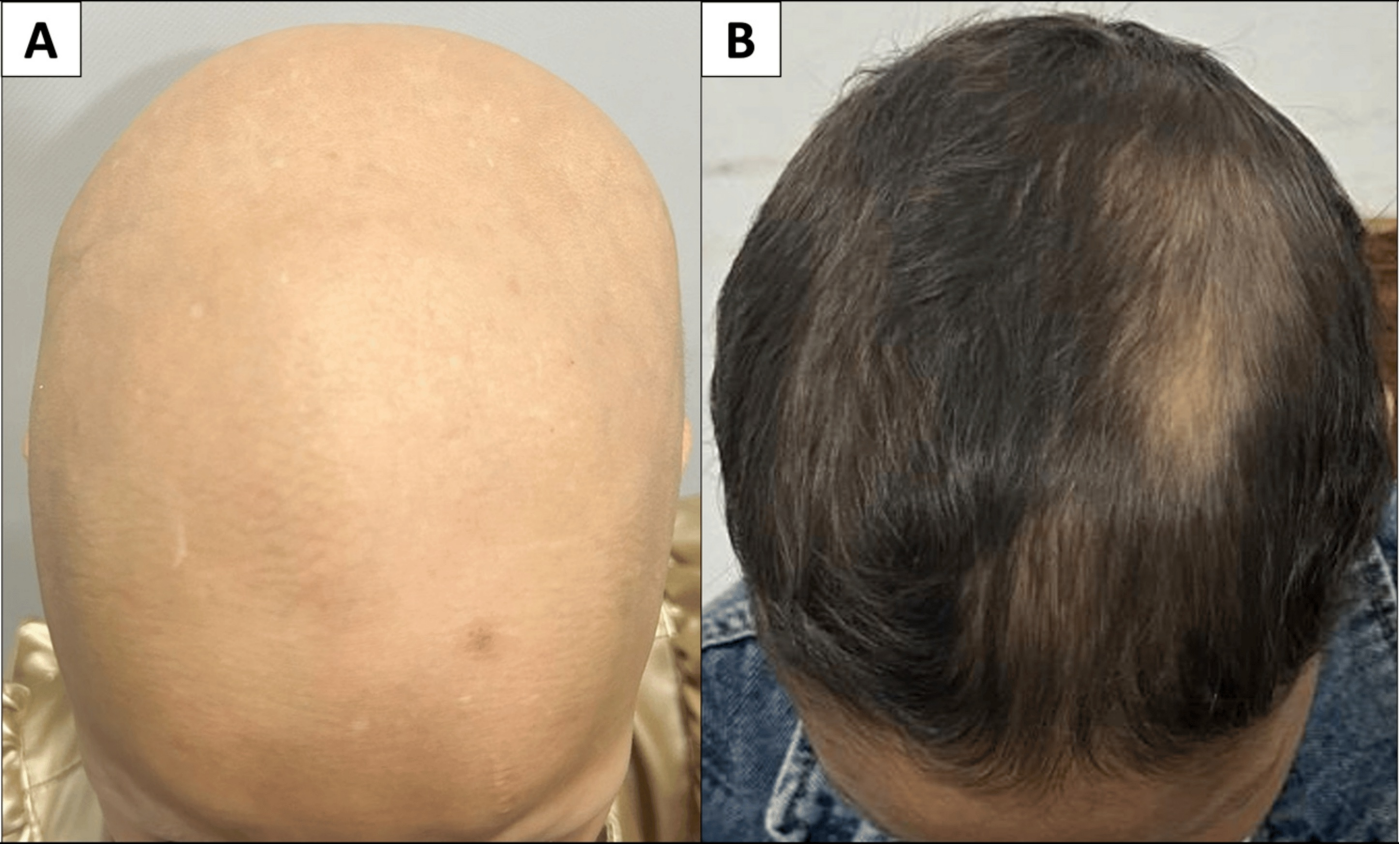
Marked clinical improvement in an eight-year-old patient with alopecia universalis following oral betamethasone mini-pulse therapy (A): Baseline presentation showing complete scalp hair loss (B): Significant regrowth (≥50%) observed after six months of treatment, with restored scalp coverage and hair density

**Figure 2:**
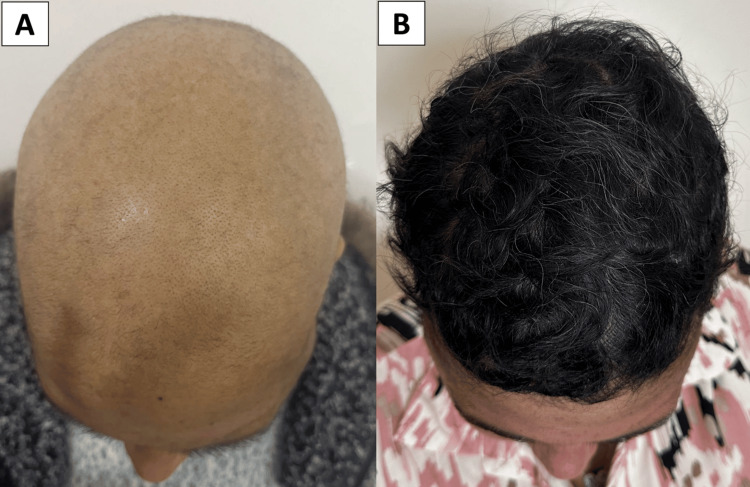
Complete hair regrowth in a 30-year-old patient with alopecia totalis following six months of oral betamethasone mini-pulse therapy (A): Baseline presentation showing total scalp hair loss (B): Marked regrowth of dense, pigmented scalp hair after treatment

Treatment resulted in significant clinical improvement. The average time to visible regrowth was 2.7 months. A ≥50% reduction in SALT (SALT-50) was achieved in 62.5% of patients (Figure 1), and complete regrowth was observed in 25% (Figure 2).

The mean SALT score decreased from 54.1% to 24.9% (p < 0.001). Eyebrow, eyelash, and body hair involvement also decreased significantly. DLQI scores improved from a mean of 5.2 to 1.8 (p < 0.001), and 77.5% of patients reported being satisfied or highly satisfied with treatment. The main outcome measures are summarized in Table 1.

**Table 1:** Main outcome measures before and after treatment with oral betamethasone mini-pulses Changes in scalp involvement (SALT score), eyebrow and eyelash loss (EBA and ELA), body hair involvement (B1-B2), and quality of life (DLQI) were assessed pre- and post-treatment. All comparisons were analyzed using the Wilcoxon signed-rank test. A p-value less than 0.05 was considered statistically significant. SALT: Severity of Alopecia Tool; EBA: Eyebrow Assessment Scale; ELA: Eyelash Assessment Scale; DLQI: Dermatology Life Quality Index

Outcome measure	Baseline	Post-treatment	p-value
SALT score (mean)	54.1	24.9	<0.001
Eyebrow involvement (EBA 0-2, %)	37.5%	22.5%	0.001
Eyelash involvement (ELA 0-2, %)	27.5%	15%	0.019
Body hair involvement (B1-B2, %)	20%	7.5%	0.026
DLQI score (mean)	5.2	1.8	<0.001

**Figure 3:**
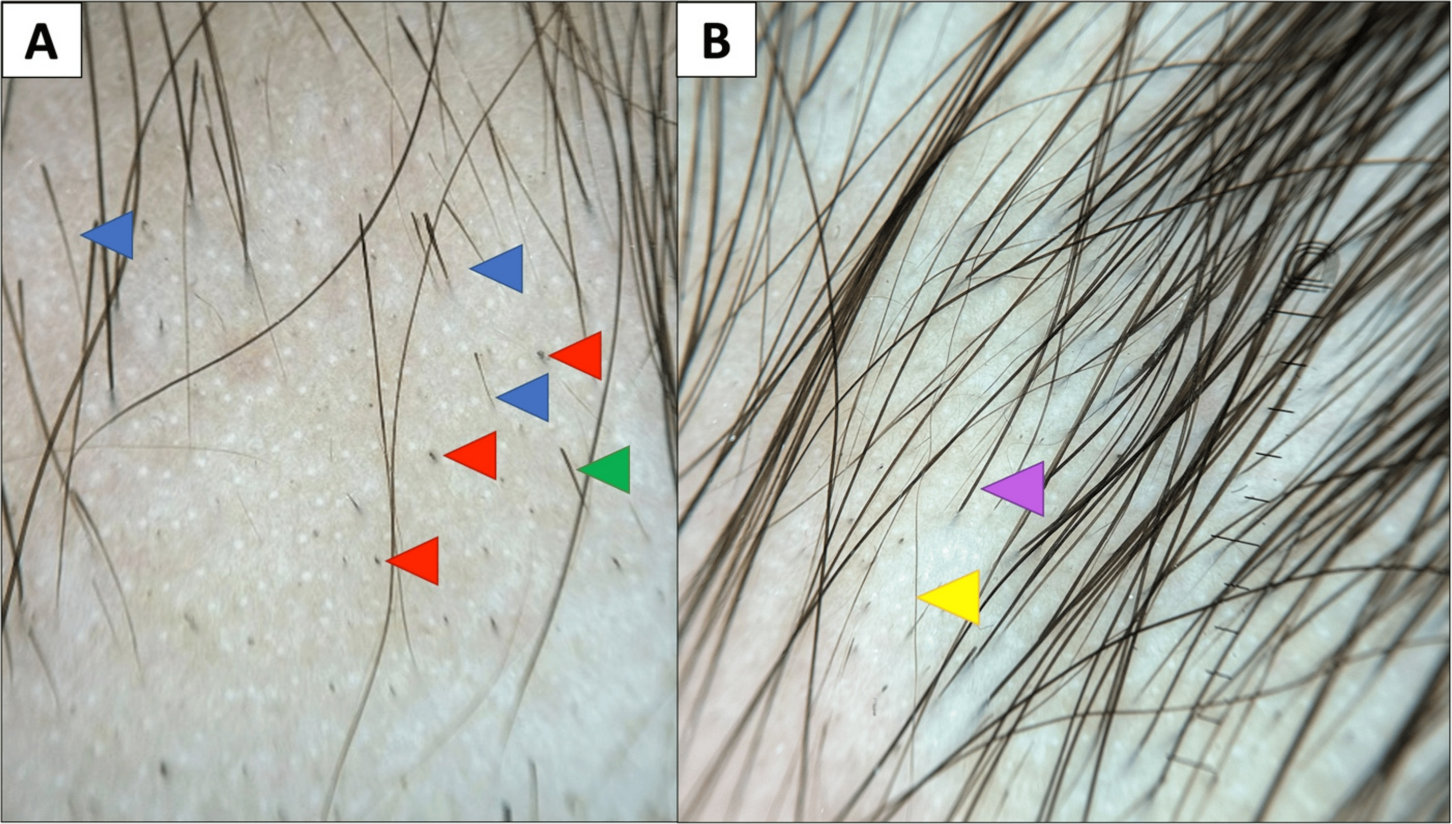
Trichoscopic evolution in a patient treated with oral betamethasone mini-pulses (A): Before treatment, showing predominance of disease activity markers: black dots (red arrows), exclamation mark hairs (blue arrows), and broken hairs (green arrow) (B): After treatment, marked predominance of regrowth indicators: vellus hairs (yellow arrow) and upright terminal hairs (purple arrow)

Trichoscopic evaluation at the end of treatment showed a marked reduction in disease activity markers. Observed trends included a decrease in black dots from 77.5% to 27.5%, exclamation mark hairs from 72.5% to 20%, and broken hairs from 62.5% to 17.5%. In parallel, signs of regrowth increased considerably: vellus hairs from 15% to 50%, upright regrowing hairs from 5% to 55%, and pigtail hairs from 5% to 17.5% (Table 2, Figure 3).

**Table 2:** Trichoscopic features before and after treatment with oral betamethasone mini-pulses Trichoscopic signs were grouped into three categories: activity (e.g., black dots, broken hairs), severity (e.g., white/yellow dots), and regrowth indicators (e.g., vellus and pigtail hairs). Percentages represent the proportion of patients in whom each feature was observed at baseline and after treatment. Dermoscopic data are descriptive; no statistical analysis was performed here.

Trichoscopic feature	Baseline (%)	Post-treatment (%)
Activity signs		
Black dots	77.5	27.5
Exclamation mark hairs	72.5	20.0
Broken hairs	62.5	17.5
Bent hairs	20.0	7.5
Pohl-Pinkus constrictions	17.5	5.0
Severity signs		
White dots	32.5	20.0
Yellow dots	17.5	7.5
Absent follicular openings	7.5	7.5
Honeycomb pigmentation	5.0	5.0
Regrowth signs		
Vellus hairs	15.0	50.0
Upright regrowing hairs	5.0	55.0
Pigtail hairs	5.0	17.5

Tolerability

The treatment was well tolerated. Adverse events occurred in 20% of patients, the most common being mild hypertrichosis (12.5%), which may have been related to topical minoxidil use, as it was consistently localized to the face and neck. Other side effects included cushingoid facies, sleep disturbances, and rosacea flare, each observed in a single patient (2.5%). No serious adverse events were reported.

Follow-up and relapse

The average total treatment duration was 9.9 months. At a mean follow-up of 10 months, 88% (22 of 25) of patients who achieved a favorable response (≥50% regrowth) maintained their results after stopping treatment, with no relapse.

Relapse was observed in 15% of patients. Half of them (7.5%) had shown poor initial response (<25% regrowth), while the remaining half had achieved satisfactory improvement (≥50% regrowth) during treatment.

Predictive factors

Statistical analysis identified several baseline factors associated with treatment outcomes. Patients with patchy AA and lower baseline SALT scores were significantly more likely to respond favorably to mini-pulse therapy. In contrast, poor response was associated with higher baseline SALT scores, rapidly progressive disease, and atopic background. Although relapse was infrequent, it was significantly more common in patients with autoimmune thyroiditis or vitamin D deficiency (Table 3).

**Table 3:** Baseline characteristics associated with treatment response and relapse Associations were analyzed using the Mann–Whitney U test for continuous variables and Fisher’s exact test for categorical variables. A favorable response was defined as ≥50% hair regrowth (SALT-50), and a poor response as <25% regrowth. A p-value less than 0.05 was considered statistically significant. AA: alopecia areata; SALT: Severity of Alopecia Tool; RP-AA: rapidly progressive alopecia areata

Baseline characteristics	Association	p-value	Statistical test
Patchy AA	Favorable response	0.046	Fisher’s exact test
Lower baseline SALT score	Favorable response	0.027	Mann–Whitney U test
Higher baseline SALT score	Poor response	0.023	Mann–Whitney U test
Rapidly progressive AA (RP-AA)	Poor response	0.007	Fisher’s exact test
Atopic background	Poor response	0.046	Fisher’s exact test
Autoimmune thyroiditis	Relapse	0.045	Fisher’s exact test
Vitamin D deficiency	Relapse	0.024	Fisher’s exact test

## Discussion

Oral corticosteroids are commonly used in the management of moderate to severe or rapidly progressing AA. According to the 2024 European consensus on the treatment of AA, systemic corticosteroids may be considered as a first-line option in adults with recent (<6 months) and active moderate to severe AA. However, their long-term use - especially in children - is not recommended due to concerns regarding tolerability and safety [5]. Oral corticosteroid mini-pulses are intermittent regimens that offer the advantage of reduced cumulative toxicity, allowing for prolonged treatment courses with improved tolerability [2].

In recent years, JAK inhibitors have emerged as a promising therapeutic class, supported by clinical trial data and increasing regulatory approvals. However, their high cost and limited accessibility remain major barriers in many low-income and developing countries [10]. In this context, oral corticosteroid mini-pulses represent an accessible and promising alternative that remains highly relevant in real-world practice.

Nevertheless, betamethasone mini-pulse therapy remains underdocumented in the literature. To date, no large-scale randomized controlled trials have evaluated its efficacy or long-term outcomes [3]. Table 4 provides a comparative summary of published studies using oral betamethasone mini-pulses for AA [11-14].

**Table 4:** Summary of studies evaluating oral betamethasone mini-pulse therapy in alopecia areata Source: Data adapted from [11-14]. SALT: Severity of Alopecia Tool

Study	n	Age range (years)	Regimen	≥50% regrowth (%)	Relapse (%)	Adverse events (%)
Khaitan et al., 2004	16	14-36	5 mg/day, 2 days/week	75	6.2	25
Deshpande et al., 2011	15	7-45	0.1 mg/kg/day, 2 days/week + short contact anthralin + topical minoxidil	73.3	13.3	13.3
Gupta et al., 2019	21	24-27	5 mg/day, 2 days/week	71.4	24	76
Asilian et al., 2021	12	16-60	3 mg, 1 day/week	Median SALT reduced from 100% to 74%	Not reported	0
Present study	40	6-50	2-4 mg/day, 2 days/week	62.5	15	20

Although study outcomes varied slightly, most reported ≥50% regrowth rates between 62.5% and 75%, with relapse rates ranging from 6.2% to 24%. Moreover, no serious adverse events were reported across all four studies; side effects were consistently mild and transient [11-14]. These results align with our findings, further supporting the potential value of this regimen in real-world practice.

Furthermore, Gupta et al. [13] demonstrated that betamethasone mini-pulses were more effective than weekly azathioprine pulses in inducing hair regrowth. In another study, Asilian et al. [14] reported that combining methotrexate with betamethasone mini-pulses led to superior outcomes compared to either agent used alone, highlighting the potential benefit of combination regimens in severe or refractory cases.

Other corticosteroids have also been used in mini-pulse regimens for AA, including prednisolone and dexamethasone. Studies using dexamethasone mini-pulses (typically 2.5-5 mg/day for two days weekly) have reported SALT-50 responses between 50% and 68%, with complete regrowth rates varying from 15% to 30% [15-17]. Prednisolone-based regimens show comparable outcomes but may carry a higher risk of side effects [18-19]. Overall, while efficacy appears similar between molecules, betamethasone offers a favorable balance between potency and safety in low-dose intermittent regimens.

Beyond efficacy comparisons, several studies have highlighted baseline characteristics that may influence the response to mini-pulse corticosteroid therapy. Kar et al. (2005) [18] reported better treatment outcomes in patients with recent disease onset, limited scalp involvement, and no history of atopy. Bajaj et al. (2008) [19] observed greater efficacy in patchy-type AA. Finally, Sánchez-Díaz et al. (2022) [16] and Lobato-Berezo et al. (2022) [17] noted reduced response rates in patients with autoimmune hypothyroidism. These trends are consistent with our findings. Nevertheless, further research is needed to better define the clinical profile of patients most likely to benefit from mini-pulse regimens, particularly when using betamethasone.

To the best of our knowledge, this is the largest cohort study to date assessing betamethasone mini-pulses in AA and the first to do so in a Moroccan cohort. Strengths of the study include the integration of clinical, dermoscopic, and quality-of-life outcomes. However, its retrospective design, limited sample size, and absence of a control group restrict the strength of interpretation. Larger controlled studies are needed to confirm these results, refine patient selection, and optimize treatment protocols.

## Conclusions

Our findings suggest that oral betamethasone mini-pulses may represent a safe and effective therapeutic option for moderate to severe or refractory AA. Good to excellent regrowth was achieved in the majority of patients, with favorable tolerance and sustained results in most responders. The use of trichoscopy provided additional non-invasive insights into disease activity and regrowth, reinforcing its value as a complementary monitoring tool.
